# Gene Expression Profiles Analyzed Using Integrating RNA Sequencing, and Microarray Reveals Increased Inflammatory Response, Proliferation, and Osteoclastogenesis in Pigmented Villonodular Synovitis

**DOI:** 10.3389/fimmu.2021.665442

**Published:** 2021-06-24

**Authors:** Yang Zhao, Jiaoyun Lv, Hongwei Zhang, Jiawei Xie, Hui Dai, Xin Zhang

**Affiliations:** ^1^ Institute of Sports Medicine, Beijing Key Laboratory of Sports Injuries, Peking University Third Hospital, Beijing, China; ^2^ Department of Laboratory Medicine, Peking University Third Hospital, Beijing, China; ^3^ Department of Immunology, School of Basic Medical Sciences, NHC Key Laboratory of Medical Immunology, Peking University, Beijing, China

**Keywords:** pigmented villonodular synovitis, gene expression profiling, inflammatory response, immune checkpoint molecules, osteoclasts, rheumatoid arthritis

## Abstract

**Background:**

Pigmented villonodular synovitis (PVNS) is a rare condition that involves benign proliferation of the synovial tissue and is characterized by severe joint destruction and high recurrence even after surgical resection. However, poor understanding of the pathogenesis limits its effective therapy.

**Method:**

In this study, gene expression profiles of six patients with PVNS, 11 patients with osteoarthritis (OA), nine patients with rheumatoid arthritis (RA) (E-MTAB-6141), and three healthy subjects (GSE143514) were analyzed using integrating RNA sequencing (RNA-seq) and microarray to investigate the PVNS transcriptome. Gene ontology, string, and cytoscape were used to determine the gene functional enrichment. Cell functional molecules were detected using flow cytometry or immunohistochemical test to identify the cell subset and function. CD14^+^ cells were isolated and induced to osteoclast to evaluate the monocyte/macrophage function.

**Results:**

The most obvious local manifestations of PVNS were inflammation, including increased immune cells infiltration and cytokine secretion, and tumor phenotypes. High proportion of inflammatory cells, including T cells, natural killer (NK) cells, NKT cells, and B cells were recruited from the blood. Th17 and monocytes, especially classical monocytes but not nonclassical monocytes, increased in PVNS synovium. An obvious increase in osteoclastogenesis and macrophage activation was observed locally. Elevated expression of MMP9, SIGLEC 15, and RANK were observed in myeloid cell of PVNS than OA. When compared with RA, osteoclast differentiation and myeloid cell activation are PVNS-specific characters, whereas T cell activation is shared by PVNS and RA.

**Conclusion:**

The transcriptional expression characteristics of PVNS showed increased immune response, cell migration, and osteoclastogenesis. Osteoclast differentiation is only observed in PVNS but not RA, whereas T-cell activation is common in inflammatory arthritis.

## Background

Pigmented villonodular synovitis (PVNS) is a mesenchymal lesion of the synovial lining of joint space, which has been classified by the World Health Organization into two types—localized (nodular) type and diffuse type, and the latter is more aggressive and invasive (1, 2). PVNS is often monoarticular and seldom involves both sides and multiple joints. Knee is the most commonly involved joint, with obvious swelling, coffee color joint puncture fluid, mild pain symptoms, and mild dysfunction. The histological morphology of PVNS revealed over-proliferation of cells, including monocyte, osteoclast-like multinucleated giant cells, and lymphocytes. In addition, local vascular plexus and hemosiderin pigmentation are abundant in PVNS tissues (3). At present, standard treatment involves surgical resection combined with radiotherapy (4). However, even after surgery, PVNS has been reported to be associated with a high recurrence rate and locally destructive process (5–7). Hence, effective alternative or complementary therapy is needed.

Given the rarity, low incidence, and limited availability of clinical samples, understanding of the pathogenesis of PVNS is poor. A detailed insight into the molecular mechanism underlying PVNS pathogenesis is critical to enhance the diagnostic accuracy and therapeutic efficiency. In this regard, transcriptome analysis contributes to a comprehensive understanding of the activated genes, pathways, and transcription factors associated with the pathogenetic molecular mechanisms, and thus, it aids in identification of the diagnostic biomarkers and therapeutic targets. However, there has been only one report on PVNS transcriptome analysis. By using complementary DNA (cDNA) array, Finis et al. demonstrated that the dominant characteristics of PVNS involve tumor progression, apoptosis inhibition, tissue invasion, and inflammation (8). This result only included bioinformatics analysis coupled with tissue hematoxylin and eosin (H&E) staining and real-time quantitative polymerase chain reaction (PCR) expression identification without any functional detection or mechanism investigation, making the study descriptive and less intriguing.

On the contrary, the current study integrated RNA sequencing (RNA-seq) and microarray analysis to identify the common specific genes, reducing the bias of technical limitations. Gene expression profiles of six patients with PVNS and six patients with osteoarthritis (OA) were analyzed using RNA-seq technology and cDNA microarray. The study aimed to determine the underlying PVNS regulatory network that would provide potential targets for PVNS diagnosis and treatment.

## Methods

### Tissue Samples

Synovial tissues obtained at the time of knee surgery performed on 6 patients with diffuse PVNS and 6 patients with OA were included in cDNA microarray experiments and RNA-sequence analysis. The diagnosis of PVNS was established by conventional histologic criteria (9). The study was conducted in accordance with the Declaration of Helsinki, and the protocol was approved by the Ethics Committee of Peking University Third Hospital (Project identification code: M2019479). All patients had signed an informed consent form.

### RNA Isolation and Amplification

Total cellular RNA of synovial tissues was isolated using an RNeasy Mini kit (Qiagen, Hilden, Germany) following the manufacturer’s instructions. The concentration of each sample was measured by NanoDrop 2000 (Thermo Scientific, USA). The quality was assessed by the Agilent2200 (Agilent, USA).

Only high‐quality RNA samples (28S:18S ribosomal RNA ratio >1.8) were selected for cDNA microarray hybridization and real‐time quantitative PCR. For amplification, cDNA synthesis was performed using a cDNA Synthesis System kit (Invitrogen), according to the manufacturer’s instructions.

### Microarray Hybridization and Gene Differential Expression Analysis

Total RNA from each sample was quantified by the NanoDrop and RNA integrity was assessed by standard denaturing agarose gel electrophoresis. For microarray analysis, Agilent Array platform was employed. The sample preparation and microarray hybridization were performed based on the manufacturer’s standard protocols with minor modifications. Briefly, mRNA was purified from total RNA after removal of rRNA (mRNA-ONLY Eukaryotic mRNA Isolation Kit, Epicentre). Then, each sample was amplified and transcribed into fluorescent cRNA along the entire length of the transcripts without 3′ bias utilizing a random priming method. The labeled cRNAs were hybridized onto the Human LncRNA and mRNA Array v2.0 (8 × 60K, Arraystar). After having washed the slides, the arrays were scanned by the Agilent Scanner G2505C.

Agilent Feature Extraction software (version 11.0.1.1) was used to analyze acquired array images. Quantile normalization and subsequent data processing were performed using the GeneSpring GX v12.0 software package (Agilent Technologies). After quantile normalization of the raw data, mRNAs that at least three of 12 samples have flags in Present or Marginal (“All Targets Value”) were chosen for further data analysis. Before gene differential expression analysis, microarray probe expression values were preprocessed (background correction, normalization, and summarization) and log2-transformed and were identified through Volcano Plot filtering. Differential expressed genes were deemed significant based on adjust p-values <0.05 and log2 fold change ≥1. The accession numbers of microarray data are GSE175626 (GEO) and d210a3 (Fast genomics).

### RNA Sequencing and Gene Differential Expression Analysis

The sequencing library of each RNA sample was prepared by using Ion Total RNA-Seq Kit v2 according to the protocol provided by manufacturer (Life technologies, USA). Briefly, poly(A)-containing mRNA was purified from 5 µg total RNA with Dynabeads (Life technologies, USA). The mRNA was fragmented using RNaseIII and purified. The fragmented RNA was hybridized and ligated with Ion adaptor. The RNA fragments were reverse-transcribed and amplified to double-stranded cDNA. Then, the amplified cDNA was purified by magnetic bead-based method, and the molar concentration was determined for each cDNA library. Emulsion PCR was performed using template of cDNA library. The Template- Positive Ion PI™ Ion Sphere™ Particles were enriched and loaded on the Ion PI™ chip for sequencing. The accession number of RNA-seq data is GSE176133 (GEO) and bfdf5e (Fast genomics).

### Identification of Differentially Expressed Genes

The DE-Seq algorithm was applied to filter differentially expressed genes for the Case and Control groups. After the significance analysis and FDR (false discovery rate) analysis [Benjamini Y, et al. Controlling the false discovery rate in behavior genetics research. Behav Brain Res. 2001 Nov 1;125(1-2):279-84.], we selected the differentially expressed genes according to the FDR threshold set at p < 0.05 and FDR <0.05. And the fold changes of any two groups are more than 2.

### Gene Enrichment Analysis

Gene ontology (GO) of differential expressed genes was carried out using the clusterProfiler package (10). GO terms are dynamically structured control vocabulary that can be applied to describe functions of genes and by which genes can be classified into three major categories, namely Biological Process, Molecular Function, and Cellular Component, and their sub-categories. P values were adjusted using the Benjamini-Hochberg method. Adjusted p-values < 0.05 were considered statistically significant. Based on the relationship of GO terms (is_a, part_of, regulates, positively_regulates, etc), GO graph of all enriched GO terms were constructed. The associated enriched GO terms in the GO graph were considered as a same subset.

Similarly, pathway analysis was used to find out the significant pathway of the differential genes according to KEGG database. Still, Fisher’s exact test followed by Benjamini–Hochberg (BH) multiple testing correction was calculated to select the significant pathway, and the threshold of significance was de- fined by *P*-value and FDR. The significant pathway was identified by P value <0.05 and FDR < 0.05.

GSEA were performed using Java software GSEA (11) (http://www.broadinstitute.org/gsea). Molecular Signatures Database (MSigDB) was employed. False discovery rate (FDR) q values were calculated using 1000 permutations, and a gene set was considered significantly enriched if its normalized enrichment score (NES) has an FDR q below 0.25.

### Protein-Protein Interaction Analysis

For protein-protein interaction analysis, STRING database (12) (http://string-db.org/) were employed. Protein-protein interaction data (V11.0) were downloaded locally. A R script was employed to construct interaction of focused genes.

### Data Visualization

The network visualization of proteins-protein interactions and GO terms relationships were carried out by Cytoscape. Heatmap of genes were drawn by Pheatmap package in R language. Venn diagrams of differential expressed genes were drawn by VennDiagram. Other graphs were drawn by R langue and ggplot2.

### Flow Cytometry Assays for Phenotype

Single-cell suspensions were stained for cell surface and intracellular markers with the following conjugated monoclonal antibodies: APC/Cyanine7 anti-human CD45 (Biolegend, catalog number: 304014), Alexa Fluor 700 anti-human CD3 (Biolegend, catalog number: 344822), PE anti-human CD56 (NCAM) (Biolegend, catalog number: 318306), FITC anti-human CD4 (Biolegend, catalog number: 317408), APC anti-human CD8a (RPA-T8) (eBioscience, catalog number: 17-0088-41), BB700 anti-human CD279 (PD-1) (EH12.1) (BD, catalog number: 566460), PE/Cyanine7 anti-human CD366 (Tim-3) (Biolegend, catalog number: 345013), PE anti-human CD152 (CTLA-4) (Biolegend, catalog number: 349906), Brilliant Violet 510 anti-human CD223 (LAG-3) (Biolegend, catalog number: 369318), PE-Cy™7 anti-human CD11b (BD Pharmingen, catalog number: 557743), FITC donkey anti-rabbit IgG (minimal x-reactivity) antibody (Biolegend, catalog number: 406403), BB515 anti-human CD25 (BD, catalog number: 564467), BV421 anti-human CD127 (BD, catalog number: 562436), APC anti-human CD183 (CXCR3) (Biolegend, catalog number: 353707), PE anti-human CD196 (CCR6) (Biolegend, catalog number: 353410), Brilliant Violet 510™ anti-human CD195 (CCR5) (Biolegend, catalog number: 359127), FITC anti-human CD14 (Biolegend, catalog number: 301804), PerCP-Cy™5.5 anti-human CD16 (BD, catalog number: 560717), Brilliant Violet 605™ anti-human CD197 (CCR7) (Biolegend, catalog number: 353224). Samples were detected with a BD FACSCanto instrument (BD Biosciences, NJ, USA), and the results were analyzed using FlowJo software V10.

### Osteoclast, Osteoblast Cultures, and Functional Assays

PVNS synovium were harvested and washed with phosphate-buffered saline (PBS), minced into small pieces (approximately 1 mm3) on ice, and enzymatically digested with 150 U/mg collagenase I (Worthington Biochemical Corporation, Lakewood, NJ, USA) for 60 min at 37°C. After digestion, the samples were sieved through a 70-µm cell strainer, and centrifuged at 300*g* for 5 min. After the supernatant was removed, the pelleted cells were suspended in red blood cell lysis buffer (Miltenyi Biotec) to lyse the red blood cells. The CD14^+^ cells were further isolated using human CD14 positive selection kit (STEMCELL Technologies, catalog number: 17858) and cultured in α-MEM in the presence of M-CSF (25 ng/ml) for 2 days then with M-CSF (25 ng/ml) and RANKL (30 ng/ml) for 10 days. Functional assessment of osteoclast formation was performed by TRAP staining.

### Real‐Time Quantitative PCR

RT-PCR was performed using the Eco Real-Time PCR System (Illumina, CA, USA) with Hieff^®^ qPCR SYBR Green Master Mix (YEASEN, Shanghai, China). Species-specific mouse primers were synthesized by Tsingke Biotech (Beijing, China) The sequences of primers used for RT-PCR are as follows (forward and reverse).

GAPDH: AGGTCGGTGTGAACGGATTTG, TGTAGACCATGTAGTTGAGGTCA; MMP9: AGACCTGGGCAGATTCCAAAC, CGGCAAGTCTTCCGAGTAGT; SIGLEC15: CGCGGATCGTCAACATCTC, GTTCGGCGGTCACTAGGTG; IL7R: CTCCAACCGGCAGCAATGTAT, AGATGACCAACAGAGCGACAG; CCL20: TGCTGTACCAAGAGTTTGCTC, CGCACACAGACAACTTTTTCTTT; TNFRSF11A: AGATCGCTCCTCCATGTAC, GCCTTGCCTGTATCACAAACTTT;

RAC2: CAACGCCTTTCCCGGAGAG, TCCGTCTGTGGATAGGAGAGC; MMP11: CCGCAACCGACAGAAGAGG, ATCGCTCCATACCTTTAGGGC; FCER1G: GTGCGAAAGGCAGCTATAACC, GGTGGTTTCTCATGCTTCAGAGT; CSF3R: GCGCGAGCAATAGCAACAAG, GTCACGATGATCTCATAGAGCTG; TGFBI: ATGACCCTCACCTCTATGTACC, CACAGTTCACAGTTACAATCCCA; COTL1: CCAAGATCGACAAAGAGGCTT, CGATGGTGGAGCCGTCATATTT; GPIHBP1: AGGTGGAAGAGGAGGAGACC, GTGAGCAGTTCTGCGTCAGGTT; FBN1: GCGGAAATCAGTGTATTGTCCC, CAGTGTTGTATGGATCTGGAGC; WNT11: GACCTCAAGACCCGATACCTG, TAGACGAGTTCCGAGTCCTTC; BMP5: AAGACTACGGAACCACGAAAGA, GGTGCAGAGGACGCTTGTTT; SPP1: GAAGTTTCGCAGACCTGACAT, GTATGCACCATTCAACTCCTCG; TFRC: GGCTACTTGGGCTATTGTAAAGG, CAGTTTCTCCGACAACTTTCTCT; CSF1: TGAGACACCTCTCCAGTTGCTG, GCAATCAGGCTTGGTCACCACA; GPC3: ATTGGCAAGTTATGTGCCCAT, TTCGGCTGGATAAGGTTTCTTC; EPHA2: TGGCTCACACACCCGTATG, GTCGCCAGACATCACGTTG; LTBP3: CCAACTGCCACCACGACTC, AGTGGGAGCGATCTCTACGG; DCSTAMP: CCTTGCCACTCCACTAAGTGT, CTCTGTGGTTGTTGCCATCTG; OCSTAMP: GCGGTTTGACAATATCTACG, AGCCTTAGAAGACAACTCAACA; PPARGC1B: TGAGCAGACCTTGACAGTGG, CTATGCTTGATGTCTGGTTTGA; CA2: ATCGACACTCATACAGCCAAGT, AAAGCATGACCATTGTTGAGGA; CD109: GTAGCATGGCAGTTCATAGTCTG, ACCACCAACTCAAAAGGCGAT; CTSK: GAGGCTTCTCTTGGTGTCCATAC, TTACTGCGGGAATGAGACAGGG; MMP7: TCGGAGGAGATGCTCACTTCGA, GGATCAGAGGAATGTCCCATACC; MMP10: TGCTCTGCCTATCCTCTGAGT, TCACATCCTTTTCGAGGTTGTAG; MMP12: GATGCTGTCACTACCGTGGG, CAATGCCAGATGGCAAGGTTGG.

After incubation at 50°C for 2 minutes, amplification reactions were performed using the following program: 95°C for 10 minutes followed by 40 cycles of 95°C for 10 s, 57-60°C for 30 s, and then a melting curve analysis from 55° C to 95°C. The relative expression level was normalized to Gapdh mRNA transcripts using the 2^−ΔΔCt^ method. Each reaction was set up in at least duplicate wells.

### Immunohistochemistry

Tissue samples from PVNS and OA patients were collected and fixed in 4% formalin for 24 h. Tissue samples were dehydrated and then embedded in paraffin. Paraffin sections (4 μm thickness) were stained with hematoxylin and eosin (H&E) for histopathology. Immunostainings were performed using the avidin–biotin–peroxidase detection method. Universal secondary antibody (Multilink; BioGenex, San Ramon, CA) was used. The sections were counterstained with hematoxylin. Anti‐human antibodies including anti–CD45, anti-CD68, anti-Ki67, and anti-desmin were purchased from CST technologies. Anti-MMP9 (catalog number: ab76003), anti-RANK (catalog number: ab12008) were purchased from Abcam Technologies, and anti-SIGLEC15 (catalog number: PA5-72765) was purchased from Thermofisher Scientific.

### Statistical Analysis

Data were expressed as mean ± standard error of the mean and were analyzed and graphed with Prism software version 8 (GraphPad, CA, USA). Statistical analysis was calculated using Student’s t test (unpaired, two tailed) and one-way of variance (with the Kruskal-Wallis test). Differences were considered statistically significant when the *p*-value was <0.05.

## Results

### Transcriptional Profiling of PVNS Using cDNA Microarray and RNA-Seq

**Figure 1:**
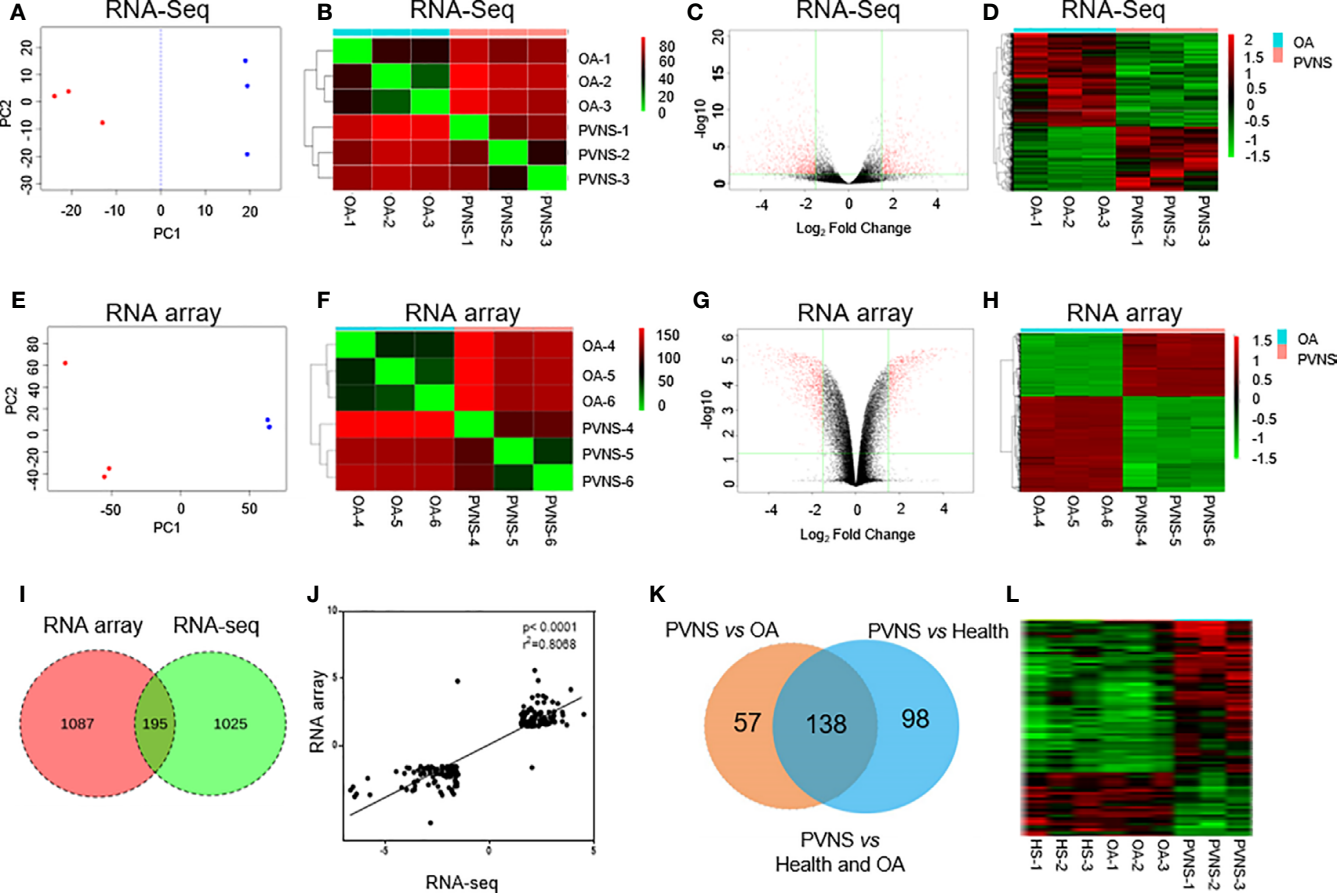
Global analysis of gene expression in pigmented villonodular synovitis (PVNS) compared to osteoarthritis (OA). **(A, E)** Principal component analysis of gene expression patterns of all genes in both PVNS and OA patient samples. Both the samples constitute two clusters. **(B, F)** Correlation heatmap of PVNS and OA transcriptomes. **(C, G)** Volcano plots of host gene expression from the RNA sequencing (RNA-Seq) analysis of PVNS and OA. **(D, H)** Heatmap of differentially expressed genes (DEGs) from both PVNS and OA samples; samples were clustered due to their similarity in gene expression. The color key indicates the relative gene expression intensity. **(I)** Venn diagram of DEGs reported in RNA-Seq and RNA microarray analyses. Only genes with q-value< 0.01, fold change >2, and fragments per kilobase of transcript per million mapped reads >1 were included. **(J)** Gene expression changes of common DEGs found by RNA-seq compared to RNA microarray. **(K, L)** Venn diagram of DEGs of PVNS *vs.* OA and PVNS *vs.* Health (GSE143514). Only genes with q-value< 0.01, fold change >2, and fragments per kilobase of transcript per million mapped reads >1 were included.

Transcriptional profiling of PVNS was analyzed using RNA microarray and RNA-seq, each analysis included three PVNS and three OA patients. In RNA-seq analysis, 28,753 human cDNA clones were detected (**Figures 1A–D**), whereas in RNA microarray analysis, sample RNA and a common reference RNA were labeled with fluorescent dye and hybridized on a microarray containing 12,762 human cDNA clones (**Figures 1E–H**)

Principle component analysis (PCA) was conducted to visualize the sample relationships, which revealed that the samples of the PVNS or OA group can be separated into two clusters according to their gene expression profile, no matter in microarray or RNA-seq Analysis. To further obtain an overview of the similarity in all samples, hierarchical cluster analysis was performed by determining the sample-to-sample distances. The samples of PVNS and OA patients were built one cluster at a time in the two analysis. Based on these results, the differentially expressed genes (DEG) in PVNS and OA groups were identified (**Figures 1A, B, E, F** **)**.

After the weakly expressed genes were filtered out and correction for multiple testing was performed, genes with q-value < 0.01, fold change (FC) > 2, and fragments per kilobase of transcript per million mapped reads (FPKM) > 1 were identified as DEGs. RNA-seq analysis identified 1,220 DEGs in the PVNS group compared to the OA group, whereas RNA microarray analysis reported 1,282 DEGs (**Figures 1C, D, G, H** **)**.

Venn diagram presentation of the DEGs in RNA-microarray and RNA-seq analyses revealed 195 common DEGs (**Figure 1I**) with consistent trend in expression changes (**Figure 1J**), confirming that these are important DEGs. Of the 195 common DEGs, 103 were upregulated and 92 were downregulated in PVNS patients compared to OA patients.

Besides OA as non-inflammatory arthritis control, three healthy synovium gene expression data (GSE143514) were also included as negative control to get specific DEG of PVNS. Of the 195 DEG of PVNS *vs* OA, 138 DEG were shared in PVNS *vs* Health, indicating the 138 genes were PVNS-specific DEG compared with healthy or non-inflammatory arthritis control (**Figures 1K, L** **)**. The following analyses were based on these common DEGs.

### Differential Expression and Pathway Enrichment Analyses

**Figure 2:**
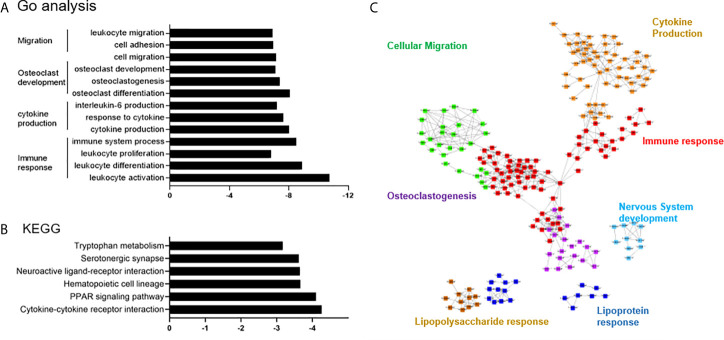
Differential expression and pathway enrichment analyses. **(A)** Functional enrichment analysis for the differentially expressed genes (DEGs) in pigmented villonodular synovitis (PVNS) sample compared to osteoarthritis (OA) control sample. **(B)** Kyoto Encyclopedia of Genes and Genomes pathway enrichment analysis demonstrating the DEGs in PVNS compared to OA. **(C)** Cytoscape plot to reflect the relationships of the Gene Ontology terms.

The DEGs in PVNS were grouped in gene ontology (GO) categories using DAVID software. Functional enrichment analysis included immune response, cytokine production, osteoclast development, and cell migration (**Figure 2A**). Analysis using cytoscape reflected the relationship between the GO terms and summarized the main biological processes in which the DEGs expressed in PVNS were involved. As shown in **Figure 2C**, the primary GO term focused on “immune effector process”, “cytokine production”, “lipopolysaccharide response”, “leukocyte migration”, “osteoclastogenesis”, and “nervous system development” (**Figure 2C**).

Kyoto Encyclopedia of Genes and Genomes pathway enrichment analysis showed that the DEGs were mainly enriched in the immune cell interactions, hematopoietic cell lineage, peroxisome proliferator-activated receptor signaling pathway, neutrophil degranulation, and several others (**Figure 2B** **)**.

Results of the global transcriptional profile analysis of the DEGs in PVNS compared to OA observations were consistent with previous reports that revealed PVNS as an invasive arthritis characterized by both inflammation and tumor phenotype (3). Although inflammation was associated with high immune response and cytokine production, cell proliferation and migration resulted in the manifestation of tumor characteristics. Furthermore, activated osteoclast and macrophage were observed in the joint. These three main aspects of the transcriptional profile in PVNS are presented and demonstrated below.

### High Immune Cell Infiltration and Increased Cytokine Secretion

**Figure 3:**
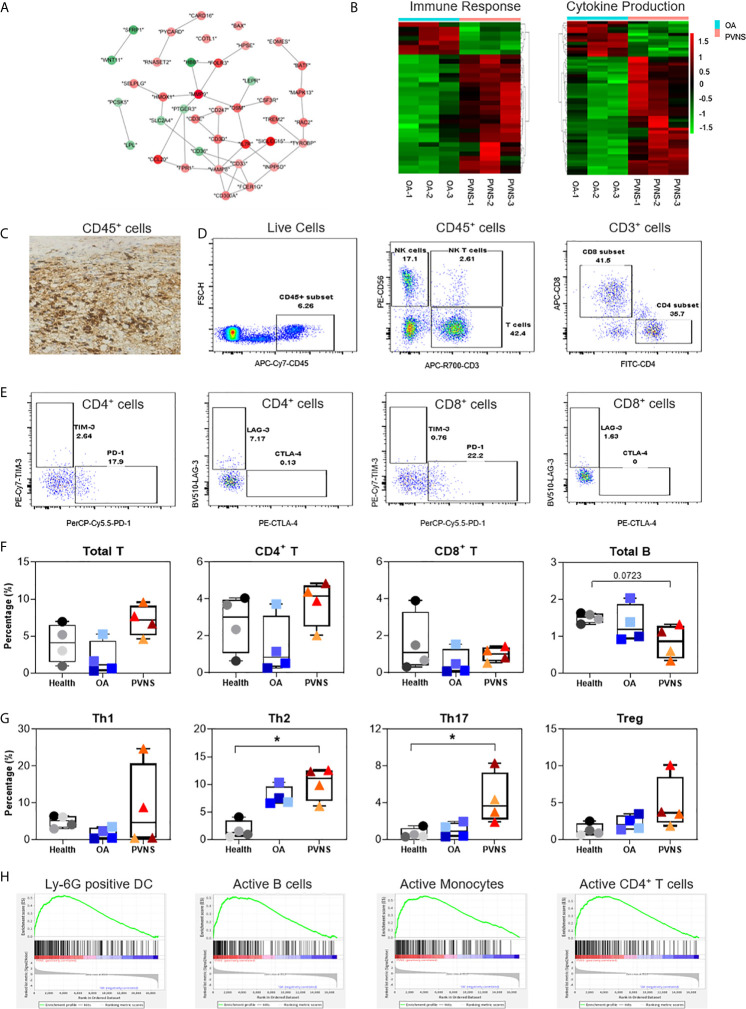
Higher immune cell infiltration and increased cytokine secretion. **(A)** Differential gene expression data of immune response between pigmented villonodular synovitis (PVNS) and osteoarthritis (OA) patient samples were analyzed using cytoscape to identify the regulated subnetworks using known disease gene association. Nodes are colored to determine the fold change. **(B)** Heatmap showing the gene expression between PVNS and OA for immune response and cytokine production subnetwork. Fold change of representative differentially expressed genes (DEGs) were also shown. **(C)** Immunohistochemical staining with CD45 specific antibody (scale bar: 100 µM). **(D, E)** Gating of flow cytometric analysis of CD45^+^ inflammatory cell populations in PVNS synovium. **(F, G)** The percentage of total T cell, CD4^+^ T cell, and CD8^+^ T cell, Treg (CD4^+^CD25^+^CD127^−^), Th1 (CD4^+^CCR7^−^CXCR3^+^), Th2 (CD4^+^CCR7^−^CCR5^+^), and Th17 (CD4^+^CCR7^−^CCR6^+^) in synovium from healthy subjects (n=4), OA patients (n=4), and PVNS patients (n=4). One-way ANOVA (Druskal-Wallis test) was applied to the multiple comparisons. *p-value< 0.05. **(H)** Gene set enrichment analysis plot depicting the enrichment of DEGs in Ly-6G, active B cells, active monocytes, and active CD4^+^ T cells in PVNS synovium.

Proliferative lesion and synovitis are considered as the critical pathogenic characteristics of PVNS. The protein-protein interaction of immune response-related genes were analyzed using string and shown in cytoscape (**Figure 3A**). Heatmaps of these genes were also generated to compare the difference in the synovium of PVNS and OA patient samples (**Figure 3B**). It is worthy of mentioning that majority of the upregulated membrane proteins were markers of immune cells, including CD3, CD6, colony-stimulating factor (CSF)2R, CSF3R, and receptor activator of nuclear factor kappa B (RANK), which is also known as TNFRSF11A, indicating the increasing immune cell infiltration in PVNS synovium.

Immunohistochemical experiments confirmed the presence of CD45^+^ inflammatory cells in the PVNS synovium, suggesting severity of the local inflammation (**Figure 3C**). Analysis of the composition of local cells using flow cytometry revealed that in addition to the CD45^−^ local fibroblasts in joints, there was a high proportion of CD45^+^ inflammatory cells, such as T cells (CD3^+^), natural killer (NK) cells (CD3^−^CD56^+^), and NKT cells (CD3^+^CD56^+^) **(** **Figure 3D** **)**. Checkpoint molecules, including programmed cell death protein 1(PD-1), T cell immunoglobulin and mucin domain 3 (TIM-3), lymphocyte-activation gene 3 (LAG-3), and cytotoxic T lymphocyte-associated protein 4 (CTLA-4), were detected on the surface of CD4^+^ and CD8^+^ T cells (**Figure 3E**).

The immune cell profiles in synovium from healthy subjects (n=4), OA patients (n=4), and PVNS patients (n=4) were analyzed, respectively. There was no difference in total T cell, CD4^+^ T cell, and CD8^+^ T cell of PVNS patients from healthy or OA controls. The number of total B cells had a trend of slightly decreasing in PVNS without significant difference (**Figure 3F**). T-cell polarization was also analyzed by percentage of Treg (CD4^+^CD25^+^CD127^−^), Th1 (CD4^+^CCR7^−^CXCR3^+^), Th2 (CD4^+^CCR7^−^CCR5^+^), and Th17 (CD4^+^CCR7^−^CCR6^+^). Th17 increased slightly (*p*<0.05) in PVNS synovium compared with healthy and OA patients. Both PVNS and OA patients had higher Th2 than healthy subjects. There was no significant difference between Th1 and Treg (**Figure 3G**)

We analyzed the related pathways or cell types of highly expressed DEGs using gene set enrichment analysis (GSEA) and found that the ones in PVNS had a relation to activated neutrophil, B cells, T cells, and monocytes. This finding suggests that the inflammatory cells activated in the joints primarily comprises immune cells from myeloid and lymphoid lineages and that the local inflammatory responses include both innate and adaptive immunity (**Figure 3H**).

### Increased Cell Proliferation and Cell Migration in PVNS

**Figure 4:**
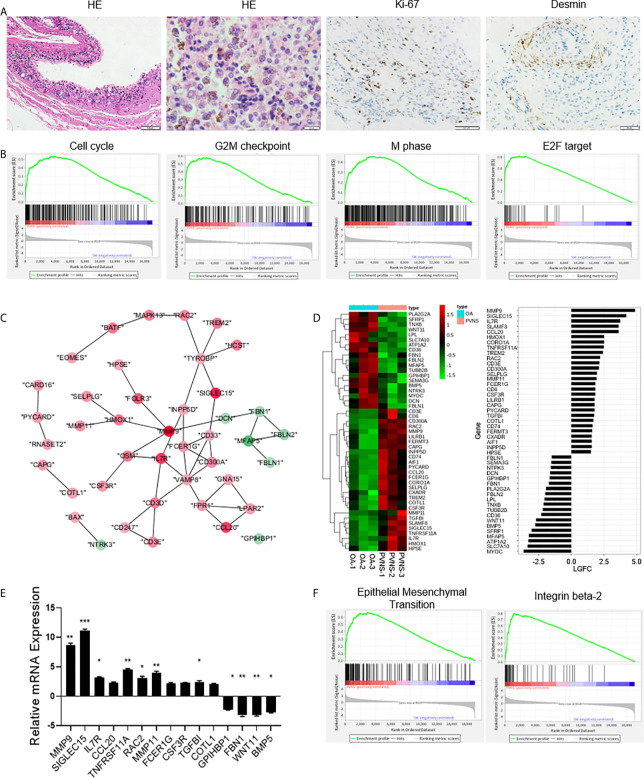
Increased cell proliferation and cell migration in pigmented villonodular synovitis (PVNS). **(A)** Hematoxylin and eosin staining and immunohistochemical staining for Ki-67 and desmin in PVNS synovium. **(B)** Gene set enrichment analysis plot depicting the enrichment of differentially expressed genes (DEGs) in cell cycle, G2M checkpoint, M phase, and E2F targets in PVNS synovium (p-value < 0.05). **(C)** Differential gene expression data of cell migration between PVNS and osteoarthritis (OA) patient samples were analyzed with Cytoscape to identify the regulated subnetworks using known disease gene association. Nodes are colored to determine the fold change. **(D)** Heatmap shows the gene expression between PVNS and OA patient samples for migration subnetwork. Fold change of representative DEG.s were also shown. **(E)** Real-time quantitative polymerase chain reaction assay for the representative DEGs to validate the result of functional enrichment analysis. Data in the bar graphs are presented as the means and standard deviations of three independent experiments. One-sample t-test was applied to the fold induction. *p-value < 0.05, **p-value < 0.01, ***p-value < 0.005. **(F)** Gene set enrichment analysis plot depicting the enrichment of DEGs in epithelial-mesenchymal transition and integrin beta-2 in the PVNS synovium (*p*-value < 0.05).

H&E staining of PVNS synovium showed obvious synovial hyperplasia and local hemosiderin pigmentation in tissues. Higher expression of Ki-67 confirmed cell proliferation in local joint (**Figure 4A**). GSEA revealed that the DEGs in PVNS were mostly associated with the cell cycle-related pathways, including G2M checkpoint and transcript factor E2F (**Figure 4B**).

Protein-protein interaction analyzed using cytoscape and heatmap of gene expression revealed that the large number of DEGs were related to cell migration, including cell adhesion, extracellular matrix, actin assembly, and cell chemotaxis (**Figures 4C, D** **)**. RT-PCR validated and verified the relative expression levels of the fifteen migration-related genes that were identified as DEGs (**Figure 4E**). Corresponding GSEA pathway analysis indicated that the epithelial-mesenchymal transition and integrin beta-2 were correlated with PVNS characteristics (**Figure 4F**).

### Increasing Osteoclastogenesis-Related Genes in PVNS

**Figure 5:**
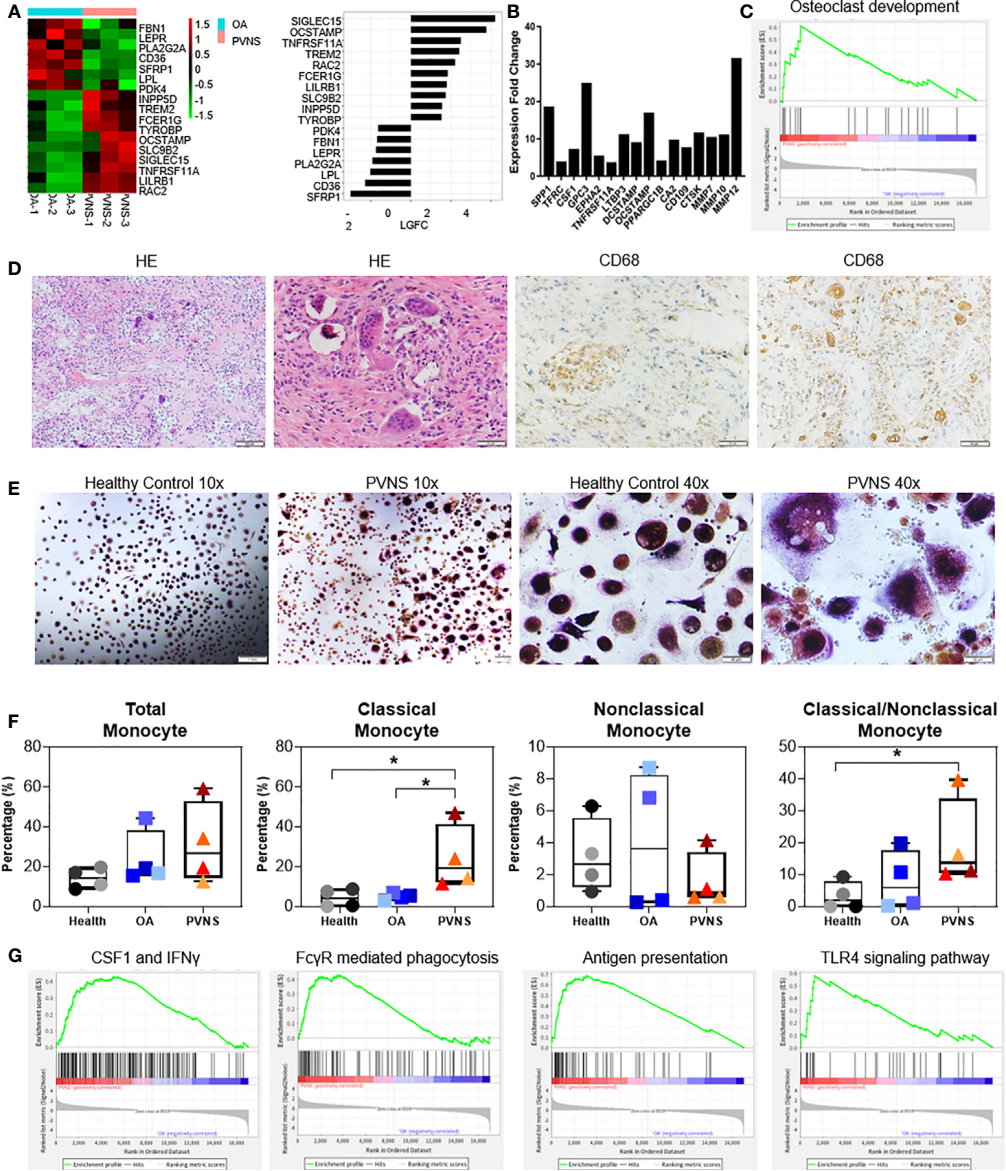
Upregulated osteoclastogenesis-related genes in pigmented villonodular synovitis (PVNS) **(A)** Heatmap showing the gene expression between PVNS and osteoarthritis (OA) for osteoclast subnetwork. Fold change of representative differentially expressed genes (DEGs) were also shown. **(B)** Real-time quantitative polymerase chain reaction assay for the representative D.E.G.s to validate the result of functional enrichment analysis. Data in the bar graphs are presented as the means and standard deviations of three independent experiments. One-sample t-test was applied to the fold induction. **(C)** Gene set enrichment analysis (GSEA) plot depicting the enrichment of DEGs associated with osteoclast development in PVNS synovium (*p*-value < 0.05). **(D)** Hematoxylin and eosin staining and immunohistochemical staining for CD68 in PVNS synovium. **(E)** CD14^+^ monocytes were isolated from PVNS or OA synovium and stimulated with macrophage colony stimulating factor and receptor activator of nuclear factor kappa B ligand for 10 days. Representative pictures of tartrate-resistant acid phosphatase staining to detect osteoclast differentiated from monocytes. **(F)** The percentage of total monocyte (CD45^+^CD11b^+^CD14^+^), classical monocytes (CD14^++^CD16^−^), and nonclassical monocytes (CD14 ^±^ CD16^+^) in synovium from healthy subjects (n=4), OA patients (n=4), and PVNS patients (n=4). One-way ANOVA (Druskal-Wallis test) was applied to the multiple comparisons. *p-value < 0.05. **(G)** GSEA plot depicting the enrichment of DEGs in CSF1 and IFN-treated, Fc receptor mediated phagocytosis, antigen presentation, and toll-like receptor 4 signaling pathway in PVNS synovium (*p*-value < 0.05).

Among the DEGs, highly upregulated genes in PVNS, such as matrix metallopeptidase 9/11 (*MMP9/11*), *TNFRSF11A*, and osteoclast stimulatory transmembrane protein (*OCSTAMP*), were closely associated with osteoclastogenesis and bone resorption (**Figure 5A**). The high expression of bone resorption-related genes was consistent with the high frequency of bone erosion in PVNS. RT-PCR demonstrated the increased expression of *OCSTAMP*, sialic acid-binding immunoglobulin-like lectin 15 (*SIGLEC15*), and *TNFRSF11A.* in PVNS compared to OA samples (**Figure 5B**).

The correlation between the highly expressed DEGs in PVNS and osteoclast were confirmed using GO term functional enrichment (**Figure 2A**) and GSEA (**Figure 5C**). H&E staining of the PVNS synovial tissues revealed the typical multinucleated giant cell morphology of the osteoclast (**Figure 5D**).

CD14^+^ monocytes purified from synovial tissue of PVNS and OA samples were made to differentiate into osteoclasts with M-CSF and RANK ligand (RANKL) treatment, respectively, for 2 weeks. Tartrate-resistant acid phosphatase-positive multinucleated cells with > 3 nuclei were considered as osteoclasts. As shown in **Figure 5E**, the number of mature osteoclasts that differentiated from PVNS was significantly higher than that from OA synovial tissue.

The immune cells from the synovium of healthy subjects (n=4), OA patients (n=4), and PVNS patients (n=4) were digested for analysis of monocytes/macrophages cell number and activation. The amount of total monocyte (CD45^+^CD11b^+^CD14^+^) had a trend of increasing in PVNS without significant difference. In monocytes, classical monocytes (CD14^+++^CD16^−^) but not nonclassical monocytes (CD14 ^±^ CD16^+^) elevated in PVNS compared with health and OA patients (*p*<0.05), indicating the increasing monocytes/macrophage were pro-inflammatory cells (**Figure 5F**).

Since macrophages are precursors of osteoclasts, the functions of macrophage were also analyzed. GSEA pathway analysis revealed activation of several macrophage functions in PVNS, such as interferon (IFN) response, Fc receptor (FcR)-mediated phagocytosis, antigen presentation, and toll-like receptor (TLR)4 signaling pathway (**Figure 5G**).

### PVNS-Specific DEG. Analysis Compared With Rheumatoid Arthritis (RA)

**Figure 6:**
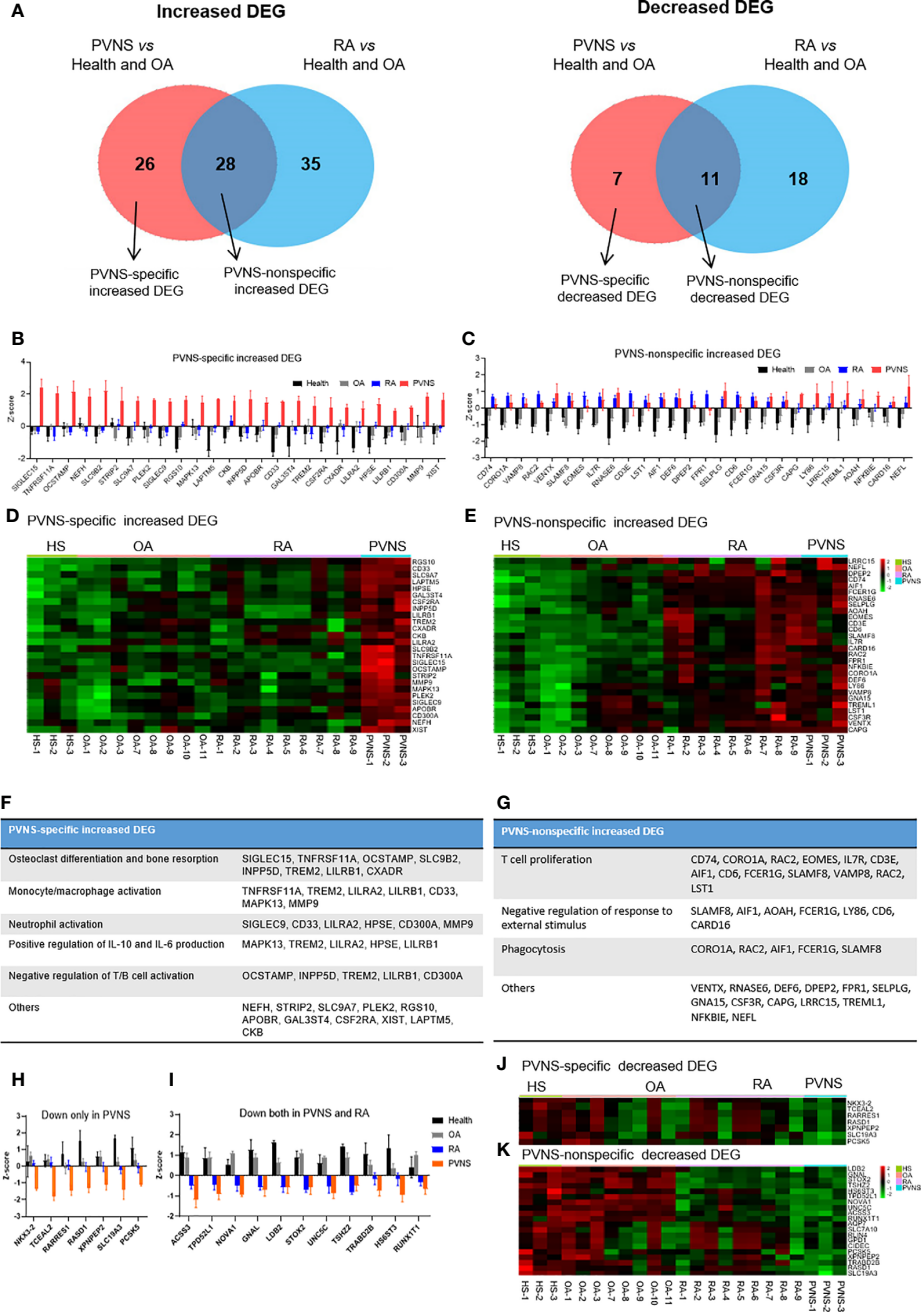
PVNS-specific DEG. analysis compared with rheumatoid arthritis (RA). **(A)** Venn diagram of increasing (left) and decreasing (right) DEGs of PVNS *vs*. OA and PVNS *vs*. Health (GSE143514). Only genes with q-value< 0.01, fold change *>*2, and fragments per kilobase of transcript per million mapped reads *>*1 were included. DEG only in PVNS were described as *“*PVNS-specific DEG*”*, and DEG shared by PVNS and RA were described as *“*PVNS-nonspecific DEG.*”* **(B, C)** Individual increasing DEG mRNA expression in healthy subjects (n=3), OA patients (n=8), RA patients (n=9), and PVNS patients (n=3) were calculated and shown by z-score. **(D, E)** Corresponding heatmap of increasing DEG in each samples. **(F, G)** Functional enrichment analysis for *“*PVNS-specific increasing DEG*”* and *“*PVNS-nonspecific increasing DEG*”*. **(H, I)** Individual decreasing DEG mRNA expression in healthy subjects (n=4), OA patients (n=8), RA patients (n=9), and PVNS patients (n=3) were calculated and shown by z-score. **(J, K)** Corresponding heatmap of decreasing DEG in each samples.

To further investigate the character of mRNA profile specific to PVNS, we expanded the control groups. Except for three healthy controls and three OA patients, another five OA patients (GSE143514), and nine rheumatoid arthritis (RA) (E-MTAB-6141) were included for analysis. Using three healthy synovium and eight OA samples as the negative controls, the increased DEG of PVNS *vs.* Health and OA and RA *vs.* Health and OA were analyzed, respectively. Among 54 increased DEG of PVNS *vs.* Health and OA, 28 genes were common in increased DEG of RA *vs.* Health and OA, defined as “PVNS-nonspecific increased DEG”. In comparison, 26 genes were only shown in PVNS but not in RA *vs.* Health and OA, described as “PVNS-specific increased DEG” (**Figure 6A**). The individual mRNA expression of 26 “PVNS-specific increased DEGs” and 28 “PVNS-nonspecific increased DEGs” were compared in HS, OA, RA, and PVNS, respectively (**Figures 6B, C** **)**. The corresponding heat maps were also shown **(** **Figures 6D, E** **)**. In “PVNS-nonspecific increased DEGs”, gene expressions were only higher in the PVNS group but not in HS, OA, RA (**Figures 6B, D** **)**. While in “PVNS-nonspecific increased DEGs”, gene expressions were higher in both PVNS and RA groups than HS and OA. There is no significant difference between PVNS and RA, indicating the 28 “PVNS-nonspecific increased DEGs” were common in inflammatory arthritis (**Figures 6C, E** **)**.

The functional enrichment of “PVNS-specific increased DEGs” and “PVNS-nonspecific increased DEGs” were explored by GO analysis, respectively. In “PVNS-specific increased DEGs”, “Osteoclast differentiation and bone resorption” is the most critical functional pathway, indicating more severe bone erosion shown in patients with PVNS compared with other arthritis and healthy controls. Besides that, “monocyte/macrophage activation”, “neutrophil activation”, and “IL-6 and IL-10 production” were also shown in “PVNS-specific increased DEGs”, suggesting that myeloid cell activation is one of the most apparent characters of synovial immune response in PVNS (**Figure 6F**). In “PVNS-nonspecific increased DEGs”, many genes enriched in “T-cell proliferation”, indicating T-cell activation is common in inflammatory arthritis such as PVNS and RA (**Figure 6G**).

As same as increased DEG, the decreased DEG of PVNS *vs.* Health and OA and RA *vs.* Health and OA were analyzed, respectively (**Figure 6A**). Seven genes only decreased in PVNS but not in RA were defined as “PVNS-specific decreased DEG” (**Figures 6H, J** **)**. In comparison, 11 genes decreased in PVNS and RA were described as “PVNS-nonspecific decreased DEG” (**Figures 6I, K** **)**. However, the functional enrichments were hard to get because of small gene numbers.

### Increased MMP9, SIGLEC 15, and RANK in PVNS Patients

**Figure 7:**
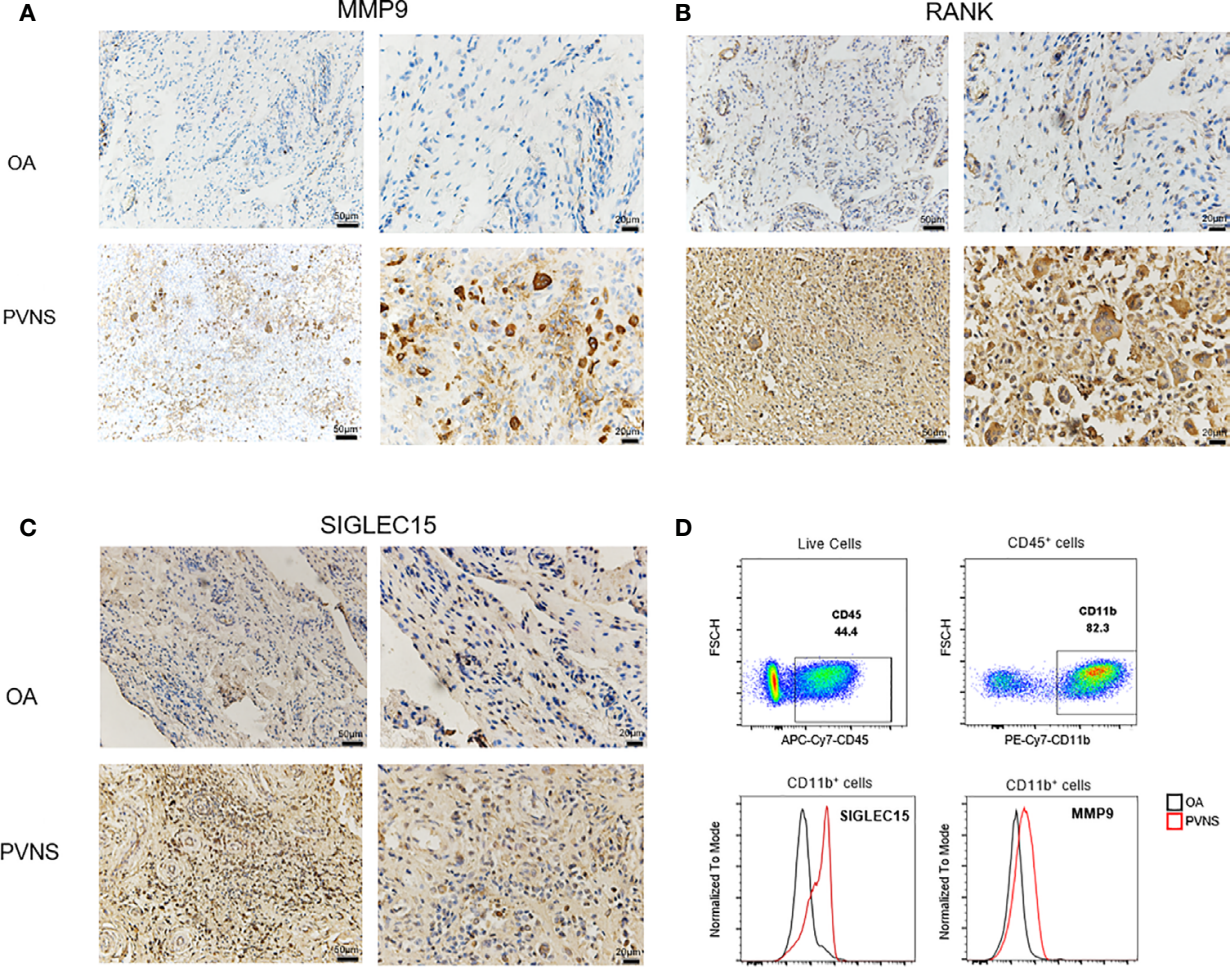
Increased MMP9, SIGLEC 15, and RANK in PVNS patients MMP9 **(A)**, SIGLEC 15 **(B)**, and RANK **(C)** expression were detected by immunohistochemical test in synovium of PVNS and OA. Label is 50μm or 20μm. **(D)** SIGLEC 15 and MMP9 expression in surface of myeloid cell (CD45^+^CD11b^+^) were detected by flow cytometry.

To confirm the gene expression levels detected by RNA-seq, the MMP9, SIGLEC15, and RANK protein expression from “PVNS-specific increased DEGs” were seen in synovial tissues obtained from PVNS patients and OA patients by immunohistochemical and flow. Higher MMP9 (**Figure 7A**), SIGLEC15 (**Figure 7B**), and RANK (**Figure 7C**) expression were shown in PVNS synovium compared with OA Elevated SIGLEC15, and MMP9 were also observed in CD45^+^CD11b^+^ myeloid cells in synovium from PVNS patients compared with OA patients detected by flow (**Figure 7D**).

## Discussion

PVNS is a rare oncological condition that has been poorly documented. In this study, RNA-seq and microarray analyses were combined to study the expression characteristics of the PVNS transcriptome. Compared to the OA patients, the transcriptome of the cells obtained from the synovium of PVNS patients showed obvious inflammation and tumor phenotypes. A large number of immune cell infiltration and increased cytokine secretion resulted in inflammation, and increased cell proliferation and migration caused manifestation of the tumor phenotype. In addition, we also observed increased osteoclastogenesis and macrophage activation locally.

Second-generation high-throughput sequencing and transcriptome microarray have become the conventional means for transcriptome analysis. Microarray is applied for the detection of known sequences, whereas sequencing can detect both known and unknown sequences in the database with higher sensitivity but quantitative deviation. The target sequence that has not undergone PCR amplification is considered more suitable for quantitative analysis. Since either of the methods have their own advantages and limitations, a combination of the two provides more accurate data. Therefore, we took samples from 6 patients with PNAS and 6 patients with OA for RNA-seq and microarray analyses. Results revealed more than 1,000 DEGs in each analysis and 195 common DEGs (**Figure 1C**). The trends in expression of these common DEGs were consistent in both the methods, indicating that the Analysis of the common DEGs could reduce the randomness and limitations of the detection methods.

Chronic inflammation in the synovial membrane of the joint tissue is considered the most important cause of tissue lesion, joint destruction, disability, and death. Thus, it is crucial to determine the key cell subsets and their activation status in the inflammatory tissues to increase our understanding of the underlying mechanism and identification of new therapeutic targets. The GO analysis of DEGs identified in the PVNS and OA patient samples showed functional enrichment in four aspects: immune response, cytokine production, migration, and osteoclast development. Of these, immune response and cytokine production were directly related to inflammation (**Figure 2A**).

Among the immune-related DEGs, the ones belonging to the mitogen-activated protein kinase (MAPK) family remain associated with a series of cellular biological processes, such as proliferation, differentiation, transcription regulation, and development. *MAPK13* regulates the transcription in response to cytokine or physical stress stimulation (13). *LY86* participates in the natural immune response to bacterial lipopolysaccharide (LPS) and cytokine production (14). *CD300A* negatively regulates the TLR signaling pathway and down-regulates the activity of NK cells and degranulation of mast cells (15, 16). Eomesodermin plays a role in the differentiation of CD8^+^ T cells and regulates the secretion of cytotoxic molecules (17, 18). Leukocyte-specific transcript 1 protein, which inhibits the proliferation of lymphocytes, can be up-regulated by LPS, IFN-gamma, and bacteria (19).

Additionally, cytokine secretion-related proteins and pathways were identified in this study. For example, TNFRSF11A, a receptor of RANKL, plays a crucial role in the activation of osteoclasts induced by RANKL and also participates in the interaction between T cells and dendritic cells (DC) (20, 21). CSF3R, a receptor of granulocyte CSF3, takes part in the proliferation, differentiation, and degranulation of the neutrophil series (22). C-C motif chemokine ligand 20 recruits the pro-inflammatory interleukin (IL)-17–producing helper T cells 17 and the regulatory T cells, and thereby contributes to the chemotaxis of DC and neutrophils (23, 24). IL-7R, a receptor of IL-7, combines with IL-2R to play a vital role in V(D)J recombination during the development of lymphocytes (25).

Apart from the inflammatory phenotype, PVNS significantly differs from the other forms of arthritis in terms of tumor phenotype. The tumor-related phenotypes observed locally in PVNS tissues mainly include increased cell proliferation and migration. Cell division cycle protein 20 (26), cyclin-dependent kinase subunit 2 (27), and E2F transcription factor 2 (28) were reported to be closely associated with cell proliferation and cell cycle. In addition, coactosin-like F-actin binding protein 1 (29), Rac family small GTPase 2, and RAC1 guanine nucleotide exchange factor mediated cytoskeleton assembly and chemotaxis (30). Another important tumor-related phenotype of the PVNS tissues is the expression of tumor checkpoint molecules by the infiltrating inflammatory cells. PD1, TIM3, LAG3, and CTLA4, expressed on the surface of CD4^+^ and CD8^+^ T cells (**Figure 3D**), indicate the occurrence of tumor immune escape phenomenon in PVNS pathogenesis.

The high expression of Siglec family of proteins, such as Siglec-15, 9, and 3, was reported in the PVNS tissues. After binding with the glycoprotein-derived sialic acid, Siglecs activate the downstream immunoreceptor tyrosine-based inhibitory motifs to play an immunosuppressed role (31). The expression of Siglec protein-encoding genes in tumor cells is considered as an important marker for tumor immune escape (32); high expression in PVNS indicates the presence of locally modified proteins with high salivary acidification, which may be a target for subsequent treatment. Furthermore, both Siglec-9 and Siglec-15 have been reported to participate in the innate immune response and remain associated with osteoclasts and osteoporosis (33, 34).

There exists a strong correlation between local inflammation and tumor phenotypes. Inflammation-mediated tumors is highly common in a variety of cancers, including skin, lung, bladder, stomach, and liver. Although the exact underlying mechanism is still unknown, chronic exposure to pro-inflammatory cytokines and growth factors under pathological conditions can transform the phenotypes of the host primary cells. For example, tumor necrosis factor-alpha-deficient mice are resistant to skin tumor development (35). Host-derived IL-1 is required for tumor invasion and angiogenesis, and the continuous activation of c-Jun N-terminal kinases promote the occurrence of chemical liver cancer (36).

Another important phenotype of the PVNS synovium is the functional activation of osteoclast. Osteoclasts, the only cell type that destroys the articular bone, are myeloid multinucleated cells induced by RANKL and CSF1. CSF1 has been shown to be a driving factor for PVNS pathogenesis (37, 38). A large number of macrophage-like synovial cells and osteoclast-like giant cells in the lesion area of PVNS (**Figure 5D**), indicating a close association between PVNS and osteoclast activation (37, 38). In our transcriptional analysis, there were multiple osteoclast activation-related genes among the highly expressed DEGs in the PVNS samples. *OCSTAMP* promotes the fusion of cells formed by osteoclasts or foreign body giant cells and participates in osteoclast-mediated bone absorption, thus playing an important role in the differentiation and functional maturation of multinucleated osteoclasts (39). CSF2R and CSF3R, receptors of CSF2 and CSF3, respectively, are considered crucial in the generation, proliferation, differentiation, and functioning of the granulocytes and macrophages (40, 41). In arthritis, MMPs and cathepsin secreted from the osteoclast, macrophage, or fibroblast take part in destruction of the joints by degrading the cartilage and extracellular matrix (42).

The small sample size is a prominent limitation of this study. Although the two methods, RNA-Seq and microarray, were combined to reduce the bias introduced when using a single method, 6 PVNS and 6 OA patients still could not fully reflect all the aspects of PVNS transcriptomic characteristics. Expansion of the sample size and addition of tissue specific data would effectively reduce these limitations.

Taken together, this study combined bioinformatics and established experiments to analyze the transcriptional expression characteristics of PVNS. The most obvious local manifestations of PVNS were increased immune response and cytokine secretion, high cell proliferation and migration, increased osteoclastogenesis activation of macrophages, and bone injury. The essential molecules of regulatory network identified in this study would provide potential targets for PVNS diagnosis and treatment.

## Conclusion

PNVS is a rare condition that involves benign proliferation of the synovial tissue. This study utilized an integrated approach involving RNA sequencing and microarray for gene expression profile analysis to minimize the bias. Obvious inflammation and tumor phenotypes were observed in PVNS. Increased cytokine secretion and Th17 and classical monocytes infiltration resulted in inflammation, and elevated cell proliferation and migration caused tumor phenotype. Increased osteoclastogenesis and macrophage activation were observed in synovium and peripheral blood of PVNS. Osteoclast differentiation is only observed in PVNS but not RA, while T cell activation is common in inflammatory arthritis.

## Data Availability Statement

The data sets presented in this study can be found in online repositories. The accession number of microarray data is GSE175626 (GEO) and d210a3 (FAST Genomics). The accession number of RNA-seq data is GSE176133 (GEO) and bfdf5e (FAST Genomics).

## Ethics Statement

The studies involving human participants were reviewed and approved by Ethics Committee of Peking University Third Hospital (Project identification code: M2019479). The patients/participants provided their written informed consent to participate in this study.

## Author Contributions

Study conception and design: HD and XZ. Acquisition of data: YZ, JL, HZ, and JX Analysis and writing: HD, YZ, JL, and XZ. All authors contributed to the article and approved the submitted version.

## Funding

This work was supported by the National Natural Science Foundation of China (grant number 81972041, 8187090401, 81700648), Beijing Natural Science Foundation (grant number 7172112, L202054, 7192221).

## Conflict of Interest

The authors declare that the research was conducted in the absence of any commercial or financial relationships that could be construed as a potential conflict of interest.

## Abbreviations

PVNS, pigmented villonodular synovitis; OA, osteoarthritis; cDNA, complementary DNA; H&E, hematoxylin and eosin; RT-PCR, real-time quantitative polymerase chain reaction; RNA-Seq, RNA sequencing; DEG, differentially expressed genes; CSF, colony stimulating factor; RANK, receptor activator of nuclear factor kappa B; PD-1, programmed cell death protein 1; LAG-3, lymphocyte-activation gene 3; TIM-3, T cell immunoglobulin and mucin domain 3; CTLA-4, cytotoxic T lymphocyte-associated protein 4; GSEA, gene set enrichment analysis; SIGLEC, sialic-acid-binding immunoglobulin-like lectin; OCSTAMP, osteoclast stimulatory transmembrane protein; MMP, matrix metallopeptidase; IL, interleukin; IFN, interferon; TLR, toll-like receptor; FcR, Fc receptor; MAPK, mitogen-activated protein kinase.
